# Impact of holiday periods on survival following an in-hospital cardiac arrest

**DOI:** 10.1016/j.resplu.2022.100238

**Published:** 2022-04-27

**Authors:** Canice Drea Persson, Therese Djärv, Maria Ygland Rödström

**Affiliations:** a Department of Medicine Solna, Karolinska Institutet, Stockholm, Sweden; b Emergency Department, Karolinska University Hospital, Stockholm, Sweden

**Keywords:** IHCA, Vacation, Cardiopulmonary Resuscitation, ROSC

## Abstract

**Introduction:**

Higher rates of mortality following an in-hospital cardiac arrest (IHCA) has been shown during nights and weekends, changes in staff density and composition has been suggested as a possible explanation. Changes in hospital staffing patterns are also common during holiday periods.

**Aim:**

To investigate whether holiday periods are associated with decreased survival following an IHCA.

**Material and methods:**

All patients ≥18 years who experienced an IHCA at Karolinska University Hospital between 2006 and 2019 were included. Patients were identified via and data was collected from the Swedish Registry for Cardiopulmonary Resuscitation. Holiday was defined as two periods, a seven-week summer period and an approximately two-week Christmas period. The primary outcome was return of spontaneous circulation (ROSC), secondary survival to hospital discharge. Logistic regression was performed to calculate odds ratio (OR) with 95% confidence intervals (CI), adjustment was done for known confounders.

**Results:**

Out of 1936 registered cases, 264 (14%) occurred during holiday periods. Patient and event characteristics were similar on holidays compared to non-holidays. Both ratio for ROSC (45% and 55%, respectively) and survival (25% and 32% respectively) was poorer during holiday periods Adjusted OR for ROSC and survival was poorer during holiday periods compared non-holiday periods (OR 0.69 [95% CI, 0.53–0.92] and OR 0.69 [95% CI, 0.49–0.96], respectively).

**Conclusion:**

Outcomes after IHCA was poorer during holiday periods compared to non-holiday periods even if patient and event characteristics was similar. Further research is needed to better understand to what degree staffing patterns and other factors contribute to the observed difference.

## Introduction

Development of resuscitative management systems have improved survival after cardiac arrest over the last decades, however survival is still low.^1^ The quality of resuscitative care and survival depends in part on the development and implementation of treatment guidelines.^2^ Previous research has found an association between the timing of an in-hospital cardiac arrest (IHCA) and survival, i.e. up to doubled odds for being discharged alive following an IHCA during office hours compared to outside office hours3., 4. and a similarly increased survival on weekdays compared to weekends.^4^ Explanatory factors observed was more witnessed arrests and shorter interval between arrest and defibrillation during office hours.3., 4. Changes in staffing patterns between daytime and night-time are common in many hospitals, both in terms of staff density and staff expertise.5., 6. Thus, differences in survival might suggest that the quality of resuscitative care varies during different times of day, and different days of the week, and could therefore at least in part be affected by such hospital staffing factors.

A potentially similar situation, with regards to changes in hospital staffing patterns, are holiday periods. Within the Swedish healthcare system, the summer holiday period is characterized by staff shortages, overcrowded hospitals, and many junior doctors with lesser experience, annually generating extensive media coverage.

In Sweden, holiday periods are by tradition relatively distinct in time. Swedish employee holiday leave benefits are regulated through the “Annual Leave Act” dated back to 1938 declaring that every employee shall be entitled to twenty-five days of annual leave in every annual leave year. Furthermore, unless otherwise agreed, annual leave dates shall be scheduled so that the employees have at least four weeks’ annual leave during the period June to August. During the following decades, a period during which a large proportion of the workforce were industrial workers, it became customary for industries to close for the month of July hence this period became known as the “industrial holidays”. Although large companies and certainly hospitals no longer adhere to such practices, it is still highly customary for Swedish employees to schedule their summer holidays sometime from the end of June to early August. Moreover, Christmas time including New Year’s Eve is a major holiday season. Therefore, Sweden offers an ideal setting for studying the effects of holiday periods on survival following an IHCA.

To the best of the authors knowledge, it is not currently known whether there are differences in survival following an IHCA during holiday periods compared to the rest of the year. Therefore, the aim of this study was to investigate whether inpatient survival following a cardiac arrest is different during summer and Christmas vacation periods compared to the rest of the year.

## Method

### Study design

This hospital-based cohort study used the Swedish Registry for Cardiopulmonary Resuscitation (SRCR)^7^ as the main source to identify all IHCAs at any of Karolinska University Hospitals between January 1, 2006 to December 31, 2019.

Karolinska University Hospital is located in Stockholm, which is home to approximately 2,000,000 people. Karolinska is one of five large hospitals and has two equally sized sites 30 km apart: Solna and Huddinge. The Solna site is a level one trauma unit, has neuro and thoracic surgery units and provides 24/7 angiography for ST-elevation myocardial infarctions. The Huddinge site includes a geriatric ward and relatively fewer intensive care unit (ICU) beds. Karolinska has about 1 300 beds, 108 000 admissions and 1.8 million patient visits yearly.

### Study population

We defined IHCA according to the SRCR8., 9. as *“a hospitalised patient who is unresponsive with apnoea (or agonal, gasping respiration) where CPR and/or defibrillation have been initiated*.” The inclusion criteria were all adult (18 years and older) patients who had an IHCA at Karolinska University Hospital. No patients or location of the IHCA were excluded. In the case of multiple IHCAs, only the first event per year was included.

#### Data collection

Registrations into the SRCR are completed in two steps online. In step one, data concerning date and time of event, age and sex of patient, time intervals from discovery to initiation of treatments, whether the event was witnessed, whether patient was monitored via electrocardiography (ECG) at time of event, place of cardiac arrest within hospital, registered first rhythm, types of treatments given, and whether the patient had return of spontaneous circulation (ROSC), is registered. In step two, data concerning co-morbidities, whether the patient was alive at hospital discharge and cerebral functioning, is registered using information from medical files.

#### Exposure

The study exposure was the timing of an IHCA within or outside holiday periods. Two holiday periods were considered. One including week 26–32 representing a 7-week summer holiday period. And one including December 20 to January 6 representing a Christmas holiday period. The choice of week numbers (according to the ISO8601 system) to define the summer holiday period is due to the fact that this system of time is widely spread in Sweden in general and used to schedule summer holidays in particular. Defining summer holidays in terms of week numbers offered higher validity compared to specific dates as these two do not correspond between different years. Defining the Christmas holiday period in terms of specific dates was arbitrary to approximate the period during which schools are closed as a proxy for potential changes in hospital staffing patterns. Data on the exposure were retrieved from the SRCR as the registered date of the event.

#### Outcome

The main outcome was ROSC, while survival to hospital discharge was the secondary outcome. The main outcome ROSC was defined as whether the patient had an own circulation with or without support when the acute resuscitative treatment ended. The secondary outcome was survival to hospital discharge, defined as whether the patient was alive when discharged from hospital. The reasoning behind these choices were that ROSC better reflects the circumstances immediately associated with the event, whereas survival to hospital discharge introduces more unknown factors as to the exact conditions surrounding an eventual death.

### Ethics

All patients surviving their IHCA were asked six months afterwards for informed consent and agreed to participate in the SRCR and on-going studies based on it. The Regional Ethical Review Board in Stockholm, Sweden approved the study, Dnr 2013/1959-31.

### Statistical analyses

Summary descriptive statistics were calculated for all patient and event variables.

Logistic regression modelling was performed to test for associations between exposure and outcomes and adjust for a priori decided potential confounders based on previous studies10., 11. including age, sex, place of cardiac arrest and first documented heart rhythm. The presence of renal insufficiency was extracted using registry data on age, sex and pre-event, usually taken at admission to hospital, plasma creatinine levels from which “estimated Glomerular Filtration Rates” (eGFR) were estimated using the revised Lund-Malmö study equation.^12^ Renal insufficiency was defined as an eGFR < 80 ml/min/1.73 m2 for patients aged 18–50 years, and eGFR < 60 ml/min/1.73 m2 for patients aged ≥ 51 years. Point estimation of odds ratios (OR) were presented with corresponding 95% confidence intervals. All model fitting was done on a complete-case basis meaning rows with missing values on any of the included variables were excluded from analysis. All statistical analyses were performed using R^13^ statistical software version, Foundation for Statistical Computing, Vienna, Austria.

## Results

Of the 1936 events included, 1672 (86%) occurred during the non-holiday period and 264 (14%) occurred during the holiday period (Table 1). Fewer IHCA occurred during holiday periods than non-holiday periods since the ten weeks represent 19% of the time but only 14% of the IHCAs occurred in the holiday period. There were only minor differences in patient and event characteristics between groups, diabetes was slightly more common (31% and 26%, respectively, Table 1), fewer arrests occurred daytime (34% and 39%, respectively, Table 1) during non-holiday. First documented rhythm was less often shockable during holidays (17% compared to 22%, Table 1). Fewer of the IHCA during non-holidays received antiarrhythmic drugs compared to holidays (10% and 16%, respectively, *p*-value 0.032, Table 1.).

**Table 1:** Characteristics of 1936 patients having a in-hospital cardiac arrest at Karolinska University Hospital 2006–2019.

	Non-holiday Number (%)	Holiday Number (%)	p-value^a^
	(*n* = 1672)	(*n* = 264)	
Age, years			0.321
Median (Q1, Q3)	72 (62, 80)	72 (63, 80)	
Sex			0.068
Female	558 (33)	102 (39)	
Male	1113 (66)	156 (60)	
Pre-existing comorbidities			
Congestive heart failure	529 (32)	79 (30)	0.949
Diabetes	444 (26)	81 (31)	0.216
Myocardial infarction	401 (24)	70 (27)	0.657
Stroke	201 (12)	39 (15)	0.418
Malignancy	472 (28)	63 (24)	0.267
Respiratory insufficiency	536 (32)	75 (29)	0.537
Renal insufficiency	1042 (62)	166 (64)	0.809
Place of cardiac arrest			0.272
Coronary Care Unit	199 (12)	40 (15)	
Intensive Care Unit	172 (10)	23 (9)	
Intermediate care unit	30 (2)	4 (2)	
Coronary laboratory	146 (9)	14 (5)	
Operating room	33 (2)	3 (1)	
Emergency department	152 (9)	29 (11)	
General ward	849 (51)	128 (49)	
Other incl Labratory, Radiology department, etc.	95 (6)	19 (7)	
Timing of cardiac arrest			0.207
Mon–Fri 8 am–8 pm	647 (39)	88 (34)	
Night/Weekend	881 (53)	145 (56)	
Witnessed	1341 (80)	207 (80)	0.984
ECG monitored	866 (52)	127 (49)	0.498
Rapid Response Team (RRT) notified	1472 (88)	231 (89)	0.573
CPR initiated before RRT arrival	1273 (76)	201 (77)	0.803
Arrival of RRT delayed (i.e. after national goal, 4 minutes)	54 (<1)	2 (<1)	
Defibrillation			
Any attempt	557 (33)	79 (30)	0.473
Defibrillation performed before RRT arrival	218 (13)	28 (11)	0.464
Delayed (after national goal, 3 minutes) defibrillation among those with shockable first rhythm	18 (5*)	1 (2*)	
First documented rhythm			0.308
Ventricular fibrillation	266 (16)	36 (14)	
Ventricular tachycardia	104 (6)	9 (3)	
Pulseless electrical activity	394 (24)	66 (25)	
Asystole	576 (34)	91 (35)	
Pharmacologic intervention			
Antiarrhythmic	275 (16)	26 (10)	0.032
Epinephrine	1140 (68)	183 (70)	0.901
Intubation	1027 (61)	161 (62)	0.820

* Percentage based on those with shockable first rhythm not the total number in column.

a *p*-value was calculated with chi^2^ or Fishers exact if <5 patients per cell.

### Return of spontaneous circulation

In all, fewer IHCA achieved ROSC during holidays compared to non-holidays (45% and 55%, respectively, Table 2). Both crude and adjusted OR for ROSC was poorer during holidays compared to non-holidays (0.69 [95% CI, 0.53–0.89] and 0.69 [95% CI, 0.53–0.92] respectively, Table2). Amount of patients achieving ROSC per month was fewer during summer months and december (Supplementary Fig. 1).

**Table 2:** Outcomes of 1936 patients having an in-hospital cardiac arrest at Karolinska University Hospital 2006–2019.

	Number of patients	ROSC Number (%)	Crude OR (95% CI)	Adjusted* OR (95% CI)	Survival to discharge Number (%)	Crude OR (95% CI)	Adjusted* OR (95% CI)
Non-holiday	1672	916 (55)	1.00 (Reference)	1.00 (Reference)	533 (32)	1.00 (Reference)	1.00 (Reference)
Holiday	264	120 (45)	0.69 (0.53–0.89)	0.69 (0.53–0.92)	65 (25)	0.69 (0.51–0.93)	0.69 (0.49–0.96)

* Adjusted for age, sex, place of cardiac arrest, first documented rhythm,

### Survival

Survival to discharge was poorer during holidays compared to non-holidays (25% and 32% respectively, Table2). Likewise, among patients achieving ROSC, survival to discharge was poorer during holidays compared to non-holidays (54% and 58%, respectively). Both crude and adjusted OR for survival was poorer during holidays compared to non-holidays (0.69 [95% CI, 0.51–0.93] and 0.69 [95% CI, 0.49–0.96] respectively, Table2).

## Discussion

The main findings of this study suggest that there is a decreased chance of ROSC and survival following an IHCA during holiday periods compared to the rest of the year. This was significant even though patient and event characteristics were similar among the two groups, indicating unknown or unmeasured factors.

There is a previously well-established “holiday-effect” of increased mortality during major holiday periods. For instance, an American study found an increase in mortality due to cardiovascular disease during the Christmas holiday period.^14^ In response, a study from Australia found a similar effect, presumably cancelling out any seasonal climactic factors since Christmas falls during summertime in the southern hemisphere.^15^ Similar reports have been published on an increased mortality following hospital admission in Taiwan during the “Chinese New Year”^16^ and in Swedish cancer-surgery performed during holiday periods.^17^ However, none of these have assessed whether the deaths were due to IHCA. Further, there is a known variation between hospitals,18., 19. but none have assessed variations within the same hospital over time.

Interestingly, we noted that fewer IHCA occurred during holiday periods than non-holiday periods since the ten weeks represent 19% of the time but only 14% of the IHCA occurred in the holiday period. Normally, fewer patients are admitted for planned care and fewer beds are open at the hospital during holiday periods, which partly could explain the difference in incidence. We are not aware of any possible differences in adherence to do not attempt cardiopulmonary resuscitation (DNACPR) orders between different time periods and unfortanely we lack such data. However, if staff density and/or experience is lower or individual workloads is higher during holidays there is a possibility that such differ.

In contrast to previous studies on night versus daytime,4., 20., 21. we found similar patient and event characteristics between holidays and non-holidays. Of certain interest was event characteristics such as witness status, ECG monitoring, start of CPR, defibrillation, and first documented rhythm. If staff during holiday periods have less experience and/or expertise it could result in less efficient management of arrests. This could potentially be indicated by a lower proportion of cases were the rapid response team (RRT) were alerted, or lower rate of CPR and defibrillation attempts prior to arrival of the RRT. However, there was no difference in these variables between holiday and non-holiday, indicating an effective crucial first step in the chain of survival during holiday periods. Therefore, our observed difference in ROSC and survival might relate to later steps in the chain of survival, such as availability and competence regarding intra- and post-arrest diagnostics, interventions and treatments. Unfortunately, we lack such data and future studies would need to carefully a prior define these things before scrutinizing all medical files and clinical schedules for clinicians at the hospital. Within this dataset we lack information on duration of CPR, in order to at least screen for any difference we used data from a previous published study,^22^ this subset included 840 patients between 2007 and 2015 who received ROSC, among them median duration of resuscitation was 13minutes during holidays compared to 10minutes during non-holidays indicating longer time to ROSC which might affect the later outcome survival.

There were no major observed differences in the prevalence of pre-existing comorbidities of the patients between non-holiday and holiday periods. However, we did not have access to burden of disease. It is for example possible that patients within similar age groups with similar comorbidities that are less symptomatic and in better general physical health are more likely to be travelling or residing in holiday houses outside of the Stockholm area during the holiday period, and therefore not included in the current study.

Compared to the proportion of all IHCA occurring during nights and weekends, the proportion of IHCA occurring during the holiday periods is relatively smaller. Therefore, any effort to identify amenable factors associated with these periods would do best in focusing on nights and weekends. Limitations include the small number of cases during holiday periods, lack of information on post-ROSC care including intensive care and limitations in treatments, environmental factors such as temperature differences, which coincide with the defined holiday period and could affect outcomes. At least for the summer holiday period previous studies have found increased mortality rates during heatwaves.^23^ Strengths of the study include a well-established Utstein-based register for IHCA data as well as stable and regular holiday/vacation practice in Sweden, setting the study in a good position to study the effect of holiday leave and IHCA.

Since this was the first study on this topic, we firstly wanted to assess if there was an association, future studies need to assess the pathway better, such as change in patient mix and or staffing which might demand access to detailed clinical rotation schedules for the whole hospital.

In conclusion, this study found a decreased likelihood of survival following an IHCA during holiday periods compared to the rest of the year. Further research is needed to better understand to what degree staffing patterns and other factors contribute to the observed difference. These findings, if verified, might indicate a need for an education or training of holiday staff.

## CRediT authorship contribution statement

**Canice Drea Persson:** Conceptualization, Methodology, Data curation, Writing – original draft. **Therese Djärv:** Conceptualization, Writing – review & editing, Supervision. **Maria Ygland Rödström:** Conceptualization, Writing – review & editing, Supervision.

## Declaration of Competing Interest

The authors declare that they have no known competing financial interests or personal relationships that could have appeared to influence the work reported in this paper.
