# Supplementary material for: Impact of holiday periods on survival following an in-hospital cardiac arrest
Source: Resusc Plus. 2022 Apr 27;10:100238. doi: 10.1016/j.resplu.2022.100238 (PMC9062336; doi:10.1016/j.resplu.2022.100238)
Supplement: Supplementary data 1 [file mmc1.docx]

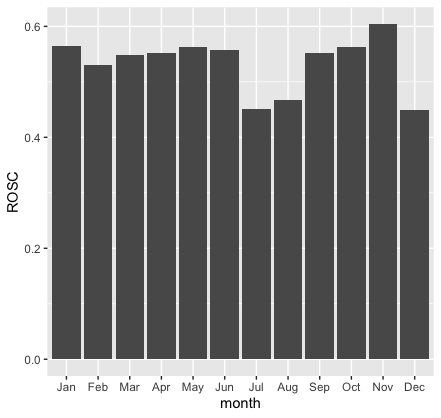


**Supplementary Fig. 1. Percentage achiving return of sponaeus circulation per month among 1936 patients having an in-hospital cardiac arrest at Karolinska University Hospital 2006–2019.**
